# Decreasing incidence of severe malaria and community-acquired bacteraemia among hospitalized children in Muheza, north-eastern Tanzania, 2006-2010

**DOI:** 10.1186/1475-2875-10-320

**Published:** 2011-10-27

**Authors:** George Mtove, Ben Amos, Behzad Nadjm, Ilse CE Hendriksen, Arjen M Dondorp, Abraham Mwambuli, Deok Ryun Kim, R Leon Ochiai, John D Clemens, Lorenz von Seidlein, Hugh Reyburn, Jacqueline Deen

**Affiliations:** 1 National Institute for Medical Research - Amani Centre, Tanga, Tanzania; 2 Joint Malaria Programme, Moshi, Tanzania; 3 Teule Hospital, Muheza, Tanga, Tanzania; 4 London School of Hygiene and Tropical Medicine, London, UK; 5 Mahidol-Oxford Research Unit, Bangkok, Thailand; 6 International Vaccine Institute, SNU Research Park, San 4-8, Nakseongdae-dong, Gwanak-gu, Seoul, Korea; 7 Menzies School of Health Research, Casuarina, NT, Australia

**Keywords:** Bacteraemia, malaria, invasive non-typhoidal salmonellosis, typhoid fever

## Abstract

**Background:**

The annual incidence and temporal trend of severe malaria and community-acquired bacteraemia during a four-year period in Muheza, Tanzania was assessed.

**Methods:**

Data on severely ill febrile children aged 2 months to 14 years from three prospective studies conducted at Muheza District Hospital from 2006 to 2010 was pooled and analysed. On admission, each enrolled child had a thin and thick blood film and at least one rapid diagnostic test for falciparum malaria, as well as a blood culture. The annual incidence of bacteraemia and severe malaria among children coming from Muheza was calculated and their temporal trend was assessed.

**Results:**

Overall, 1, 898 severe falciparum malaria and 684 bacteraemia cases were included. Of these, 1, 356 (71%) and 482 (71%), respectively, were from the referral population of Muheza. The incidence of falciparum malaria and all-cause bacteraemia in Muheza decreased five-fold and three-fold, respectively, from the first to the fourth year of surveillance (p < 0.0001). During this period, the median ages of children from Muheza admitted with severe malaria increased from 1.7 to 2.5 years (p < 0.0001). The reduction in all-cause bacteraemia was mainly driven by the 11-fold decline in the incidence of non-typhoidal salmonellosis. The annual incidences of *Haemophilus influenzae *and pneumococcal invasive bacterial infections decreased as well but were much fewer in number.

**Conclusions:**

These results add to the growing evidence of the decline in malaria associated with a decrease in non-typhoidal salmonellosis and possibly other bacteraemias. Malarial prevention and control strategies may provide a greater benefit than the mere reduction of malaria alone.

## Background

Community-acquired bacteraemia causes significant morbidity and mortality among children in sub-Saharan Africa. A recent comprehensive review that included 43, 130 children admitted to African hospitals with a blood culture, found that 3, 527 (8%) had a bloodstream infection [1]. Epidemiological observations suggest that strategies that control malaria also decrease the rates of bacterial infections. Firstly, a study in The Gambia showed that the use of insecticide-treated bed nets reduced overall child mortality to an extent greater than could be attributed to malaria alone [2]. Secondly, a trial of the RTS, S/AS01E malaria vaccine in Tanzanian and Kenyan children showed not only reduced rates of malaria in the intervention compared to the control group, but decreased incidence of pneumonia and gastroenteritis as well [3,4]. Thirdly, a four-year intensive malaria control programme on Bioko Island, Equatorial Guinea was associated with a decline in all-cause child mortality [5]. Fourthly, deployment of anti-malaria interventions in Zanzibar was associated with a substantial decrease in crude childhood mortality between 2002 and 2005 [6]. That malaria increases the risk of bacteraemia has been especially observed for invasive non-typhoidal *Salmonella *(NTS) infections. NTS, mainly *Salmonella typhimurium *and *Salmonella enteritidis*, are a leading cause of bacteraemia in African children [7-19]. In contrast to industrialized countries where NTS is usually associated with a self-limited gastroenteritis [20], invasive disease frequently occurs in sub-Saharan Africa with case fatality rates of 4 to 27% among hospitalized children [12,15,17,21].

The common occurrence of NTS septicaemia during malaria outbreaks was first reported in British Guiana in the 1920s [22]. Duggan and Beyer [23] suggested an association between invasive salmonellosis and malaria in Nigerian children. In 1987, Mabey *et al *[7] found that young Gambian children with NTS invasive infection were more anaemic and more likely to have evidence of recent malaria than were children of the same age with other forms of septicaemia. In 1999, Berkley *et al *[24] proposed that an acute episode of malaria predisposes to bacteraemia and that the latter contributes to the clinical picture of severe malaria. In Malawi, studies showed an association between NTS bacteraemia and severe anaemia [13,18,21]. In Kenya, Brent *et al *[17] found that three quarters of NTS patients with anaemia had evidence of either current or recent malaria.

During the last decade, the intensity of malaria transmission has decreased in parts of sub-Saharan Africa [25-28]. In The Gambia, Mackenzie *et al *reported lower rates of invasive NTS infection in children associated with reductions in malaria [29]. Most recently, researchers in Kenya showed that the sickle cell trait decreases the risk for invasive bacterial disease due to its known protection against malaria [30]. Using data from fever surveillance studies in children admitted to a rural district hospital in north-eastern Tanzania from 1 June 2006 to 31 May 2010, the annual incidence and temporal trend of malaria and community-acquired bacteraemia was assessed.

## Methods

**Figure 1:**
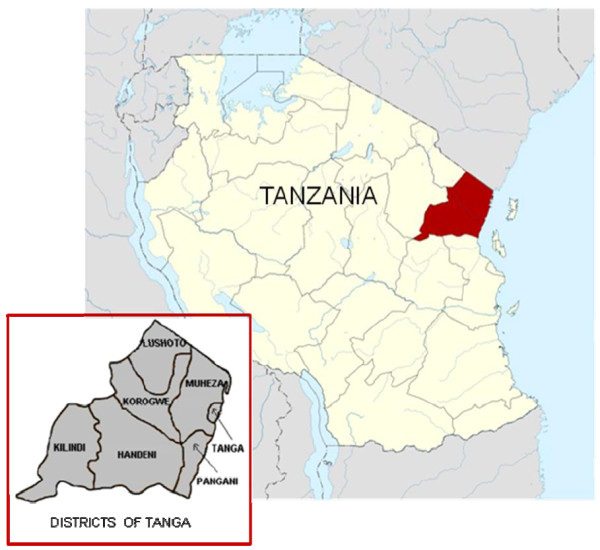
**Location of Tanga region in Tanzania, East Africa (Modified from: Wikipedia, http://en.wikipedia.org/wiki/Tanga_Region, accessed 15 August 2011)**.

The study site consisted of two paediatric in-patient wards of the Teule District Hospital in Muheza, north-eastern Tanzania. Each of the wards has 35 beds and together receives 6, 000 child admissions per year. Teule's referral base is the Muheza district (including Mkinga, which was split-off from Muheza in 2007) but the hospital accepts patients from all over the Tanga region. Tanga, one of 26 regions of Tanzania, has an area of 26, 808 square kilometres and is divided into the following districts: Lushoto, Korogwe, Muheza (and Mkinga), Tanga, Pangani, Handeni, and Kilindi (Figure 1). First-level medical care is provided by primary health centres and each district (except the newly formed Mkinga district) has a hospital. The Bombo referral hospital is located in the coastal town of Tanga (40 kilometres east of Muheza). Tanga region is endemic for *P. falciparum *malaria. In 2007, 7% of antenatal clinic attendees [31] and 4% of hospitalized febrile children [32] were HIV positive. The Tanzanian Expanded Programme of Immunization includes the following: Bacille Calmette-Guérin, live oral polio, diphtheria-whole cell pertussis-tetanus-hepatitis B and monovalent measles vaccines for children, as well as supplemental tetanus toxoid vaccine for women of child-bearing age. In Tanzania, immunization against *Haemophilus influenzae *type b (Hib) had just started in March 2009 and there are plans to introduce pneumococcal vaccine [33].

Malaria diagnostic data and blood culture results from prospective studies of febrile children undertaken at the Teule paediatric wards from 2006 to 2010 were pooled and analysed [32,34,35]. The first study was conducted from June 2006 to May 2007 and enrolled children aged two months to < 15 years with a history of fever within the previous two days admitted during study hours [32]. Shortly thereafter, a drug trial for the treatment of severe falciparum malaria was implemented [34]. In the trial, malaria testing and blood culture were performed on febrile children of the same age group admitted with clinical signs of severe malaria, including coma (Blantyre coma score ≤ 2 for children less than two years or a Glasgow coma score ≤10 for older children), prostration (inability to sit unsupported for children over nine months or to drink/breastfeed), convulsions, shock (temperature gradient in the lower extremities and delayed capillary refill of > 3 seconds or systolic blood pressure < 70 mm Hg), respiratory distress (manifested as nasal alar flaring, costal indrawing/recession, use of accessory muscles), desaturation (oxygen saturation less than 90%), suspected severe acidosis (deep breathing), hypoglycaemia (blood glucose level of < 3.0 mmol/litre), severe anaemia (haemoglobin < 5 g/dl) with respiratory distress, and severe jaundice. Finally, a second fever surveillance study was implemented from March 2008 to May 2010 to primarily assess the burden of typhoid fever [35]. The study included febrile children of the same age group admitted during study hours using the same criteria of clinical severity with the addition of neck stiffness or bulging fontanel and fever of three or more days. In all three studies, children with an obvious non-infectious cause for admission such as trauma, surgery or known malignancy were excluded. Prior to the start and during the course of the studies, emergency triage and management were implemented [36]. Treatment and referral were provided according to Tanzanian national guidelines.

The point-of-care and laboratory investigations conducted are described in detail elsewhere [32,34,35]. Each child had at least one rapid diagnostic test (RDT) for *P. falciparum *malaria using a PfHRP-2 based (Paracheck™, Orchid Biomedical, Mumbai, India or Parahit™, Span Diagnostics, Surat, India) and/or pLDH based (OptiMAL-IT, DiaMed AG, Cressier, Switzerland) kits. From each child, thin and thick blood films were prepared, Giemsa-stained and read by experienced laboratory technicians. At least 100 high-power microscopic fields of the thin film were examined to exclude the diagnosis of malaria. One to 10 ml of blood (depending on body weight) were collected from each child and inoculated into a BactALERT™ Paediatric-fan bottle (bioMérieux, Marcy l'Etoile, France) and incubated in the BacT/ALERT 3D automated microbial detection system. Blood cultures were processed according to standard methods. The laboratory participated in a bacteriology external quality assurance programme organized by the Network for Surveillance of Pneumococcal Disease in the East African Region http://www.netspear.org and identified unknown samples provided by a nearby laboratory as part of the College of American Pathologists external quality assurance programme. Bacterial identifications were independently confirmed for *Streptococcus pneumoniae *and *Haemophilus influenzae *isolates by the KEMRI/Welllcome Trust Centre for Geographic Medicine, Kilifi, Kenya and for some of the Salmonellae isolates by the Queensland Health Forensic and Scientific Services, Brisbane, Australia.

Each study used its standard case record form that documented demographic details including age and residence, clinical information, as well as point-of-care and laboratory results. Data analysis was performed using Stata software (version 11.0). Fever was defined as history of a rise in body temperature as recalled by a care-giver or presence of axillary temperature ≥37.5°C on presentation. Bacteraemia was defined as fever with isolation of pathogenic bacteria from blood culture. One blood culture was performed on admission. For the purpose of this analysis, severe falciparum malaria was defined as fever with a positive RDT or presence of asexual malaria parasites on the blood film and at least one clinical sign of severe malaria as described above. A severe febrile illness was defined as fever with at least one severity sign as described above with the addition of neck stiffness, bulging fontanel and fever of three or more days.

**Figure 2:**
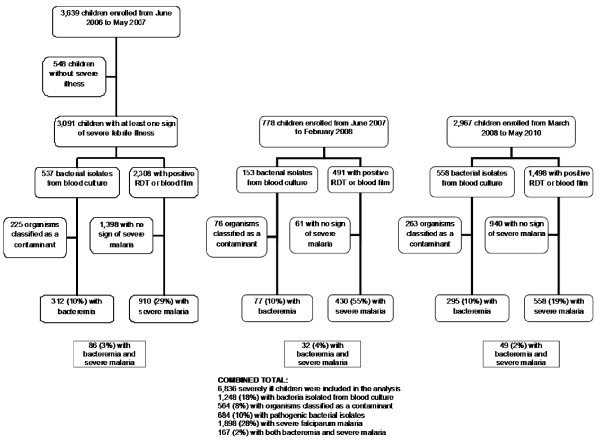
**Assembly of data**.

To apply consistent criteria for inclusion across the studies, we excluded children without signs of severe illness in the first study from the analysis (Figure 2). To ensure a valid denominator for incidence estimates, children with falciparum malaria and bacteraemia were classified by district. The annual incidence of bacteraemia and severe malaria among children coming from the referral population (Muheza, including Mkinga) was calculated per 100, 000 children under 15 years of age. The number of cases residing in the referral population was used as the numerator and the population under 15 years of age for each year, projected from the 2002 census [37], as the denominator. The referral population figures used as the denominator were 121, 465, 122, 847, 124, 322, 125, 819 children for 2006, 2007, 2008, and 2009, respectively. The chi-square test for trend in counts was used to evaluate the temporal trend of the incidence of malaria and bacteraemia [38]. The median ages of children with severe falciparum malaria at one-year intervals was assessed for temporal trend using the Jonckheere-Terpstra test, a non-parametric test using the R function (The R Foundation for Statistical Computing, version 2.13.0, Vienna, Austria) [39].

The three studies (from which the pooled data was obtained) were conducted according to the principles expressed in the Declaration of Helsinki. Written informed consent was obtained from the parent or guardian of each eligible child prior to his/her participation. The three studies were approved by the Tanzania Medical Research Coordinating Committee. In addition, the first febrile illness study was approved by the London School of Hygiene and Tropical Medicine (UK), the severe malaria drug trial was approved by the Oxford Tropical Research Ethics Committee (UK) and the surveillance study to assess the burden of typhoid fever was approved by the International Vaccine Institute - Institutional Review Board (Republic of Korea).

## Results

During the study period from June 2006 to May 2010, a total of 6, 836 severely ill febrile children between 2 months and < 15 years old who participated in the study were included in the analysis: 3, 091, 778 and 2, 967 from the first, second and third studies, respectively (Figure 2). 5, 281 (77%) of 6, 836 were from the referral population of Muheza (including Mkinga) and their characteristics, by study group, are shown in Table 1. Bacteria were isolated from the blood of 1, 248/6, 836 (18%), of which 564 (8%) were considered as likely contaminants. Each of the 684 (10%) children with bacteraemia had only a single organism isolated from their blood culture. Of the 6, 836 children, 1, 898 (28%) had severe falciparum malaria. Both bacteraemia and severe malaria were present in 167 (2%).

**Table 1:** Characteristics of the children in the three study groups

	1^st ^study	2^nd ^study	3^rd ^study	Total
**Inclusive dates of enrolment**	June 2006 to May 2007	June 2007 to February 2008	March 2008 to May 2010	June 2006 to May 2010
**Time period**	12 months	9 months	27 months	4 years
**No of children with at least one sign of severe illness**	3, 091	778	2, 967	6, 836
**No (%) from Muheza**	2, 447 (79)	448 (58)	2, 386 (80)	5, 281 (77)
**- Mean (Standard deviation) age**	2.2 (2.1)	2.3 (2.2)	2.7 (2.6)	2.4 (2.3)
**- No (%) female**	1, 157 (47)	217 (48)	1, 048 (44)	2, 422 (46)

Of the 6, 836 children included in the analysis, 4, 023 (59%) were > 2 months to 2 years, 2, 125 (31%) were > 2 years to 5 years and 688 (10%) were > 5 years of age. We ranked the pathogenic bacterial isolates according to frequency, by age group (Table 2). Overall and among children less than five years of age, NTS was the principal organism, comprising 34% of the invasive pathogens. Other commonly isolated bacterial pathogens were Hib, *S. pneumoniae*, and *Escherichia coli*. *Salmonella typhi *was the most common isolate among those over 5 to 14 years of age.

**Table 2:** Number (%) of bacterial isolates ranked* according to frequency by age group, 1 June 2006 to 31 May 2010

	No (%) among > 2 m to 2 y (n = 4, 023)	Rank	No (%) among > 2 y to 5 y (n = 2, 125)	Rank	No (%) among > 5 y to < 15 y (n = 688)	Rank	Total no (%) (n = 6, 836)	Overall rank
**Gram-positive**								
- *Streptococcus pneumoniae*	55 (13)	**4**	20 (12)	**3**	10 (13)	**2**	85 (12)	**3**
- beta haemolytic Streptococci, Group A & C	18 (4)	**5**	5 (3)	**7**	2 (3)	**6**	25 (4)	**7**
- *Staphylococcus aureus *	16 (4)	**6**	7 (4)	**6**	9 (11)	**3**	32 (5)	**6**
**Gram-negative**								
- *Salmonella typhi*	5 (1)	**7**	21 (13)	**2**	33 (42)	**1**	59 (9)	**5**
- *Nontyphoidal salmonella species *	168 (38)	**1**	58 (35)	**1**	6 (8)	**5**	232 (34)	**1**
- *Haemophilus influenzae (type B)*	69 (16)	**2**	16 (10)	**4**	7 (9)	**4**	92 (14)	**2**
- *Escherichia coli*	68 (16)	**3**	9 (6)	**5**	2 (3)	**6**	79 (12)	**4**
- *Acinetobacter species *	4 (1)	**8**	5 (3)	**7**	0 (0)	**8**	9 (1.3)	**8**
Other	5 (1)		21 (13)		33 (42)		59 (8.6)	
**All pathogenic bacteria**	**440 (11)**		**165 (8)**		**79 (11)**		**684 (10)**	

**Contaminants**	**382 (9)**		**140 (7)**		**42 (6)**		**564 (8)**	

* Rank (by age group and overall) was the same for organisms with the same frequency.

**Figure 3:**
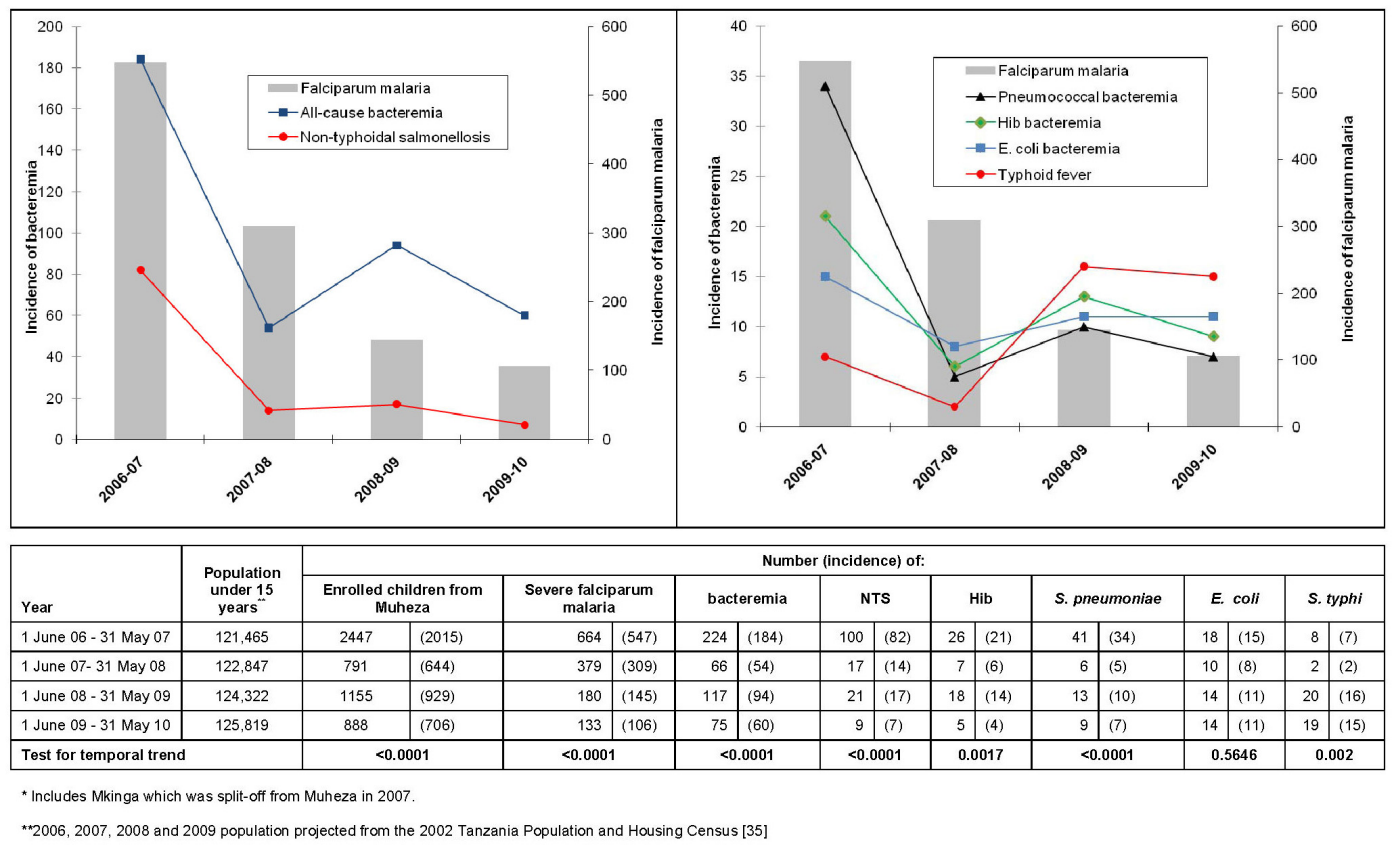
Annual incidence (per 100, 000 children under 15 years of age) of severe falciparum malaria and bacteremia in Muheza*.

The children with falciparum malaria and bacteraemia were classified by district (Table 3). Of the overall 1, 898 severe falciparum malaria and 684 bacteraemia cases, 1, 356 (71%) and 482 (71%), respectively, were from the referral population of Muheza (including Mkinga). The incidence of falciparum malaria and bacteraemia per 100, 000 children under 15 years of age in Muheza was plotted for each year of surveillance (Figure 3). The incidence of severe falciparum malaria and all-cause bacteraemia decreased five-fold and three-fold, respectively, from the first to the fourth year of surveillance (p < 0.0001). The median age of the children from Muheza admitted with severe malaria during each year of surveillance was compared (Table 4). Over the four-year period, the median age increased from 1.7 to 2.5 years (p < 0.0001). As shown in Figure 3, the reduction in all-cause bacteraemia was mainly driven by the 11-fold decline in the incidence of non-typhoidal salmonellosis from 82 to 7 cases/100, 000 children/year from the first year to the fourth year of surveillance (p < 0.0001). The annual incidences of Hib and pneumococcal invasive bacterial infections had statistically significant declines (from 21 to 4 cases/100, 000 children/year for Hib and 34 to 7 cases/100, 000 children/year for pneumococcal infections, p < 0.001). The incidence of *E. coli *bacteraemia did not change (p = 0.56) whereas that of typhoid fever increased from 7 cases/100, 000 children/year during the first year to 15 during the fourth year of surveillance (p = 0.002).

**Table 3:** Number (%) of children with bacteraemia and severe falciparum malaria by residence, 1 June 2006 to 31 May 2010

District (population under 15 years*)	Number (%) with severe falciparum malaria	Number (%) with bacteraemia						
		All pathogenic	NTS	Hib	*S. pneumoniae*	*E. coli*	*S. typhi*	*S. aureus*
**Referral population:**								
Muheza** (124, 322)	1, 356 (71.4)	482 (70.5)	147 (63.4)	56 (60.9)	69 (81.2)	56 (70.9)	49 (83.1)	22 (68.8)

**Non-referral population:**								
Lushoto (218, 957)	1 (0.1)	0 (0)	0 (0)	0 (0)	0 (0)	0 (0)	0 (0)	0 (0)
Korogwe (118, 460)	61 (3.2)	18 (2.6)	9 (3.9)	0 (0)	4 (4.7)	2 (2.5)	0 (0)	2 (6.3)
Tanga (98, 177)	10 (0.5)	6 (0.9)	2 (0.9)	1 (1.1)	0 (0)	0 (0)	2 (3.4)	0 (0)
Pangani (18, 292)	10 (0.5)	3 (0.4)	1 (0.4)	1 (1.1)	0 (0)	0 (0)	0 (0)	1 (3.1)
Handeni (123, 762)	374 (19.7)	151 (22.1)	63 (27.2)	27 (29.3)	8 (9.4)	21 (26.6)	7 (11.9)	6 (18.8)
Kilindi (74, 228)	1 (0.1)	0 (0)	0 (0)	0 (0)	0 (0)	0 (0)	0 (0)	0 (0)
District not recorded	85 (4.5)	24 (3.5)	10 (4.3)	7 (7.6)	4 (4.7)	0 (0)	1 (1.7)	1 (3.1)

**Total Tanga region (776, 198)**	1, 898 (100)	684 (100)	232 (100)	92 (100)	85 (100)	79 (100)	59 (100)	32 (100)

*2008 population projected from the 2002 Tanzania Population and Housing Census (Tanzania National Bureau of Statistics 2006).

**Includes Mkinga which was split-off from Muheza in 2007.

**Table 4:** Median age in years of children from Muheza admitted with severe falciparum malaria during each year of surveillance

Time period (number of children)	Median age in years N = 1, 356
**1 June 2006 - 31 May 2007 (N = 664)**	1.7
**1 June 2007 - 31 May 2008 (N = 379)**	2.0
**1 June 2008 - 31 May 2009 (N = 180)**	2.4
**1 June 2009 - 31 May 2010 (N = 133)**	2.5

**Test for temporal trend**	< 0.0001

**Figure 4:**
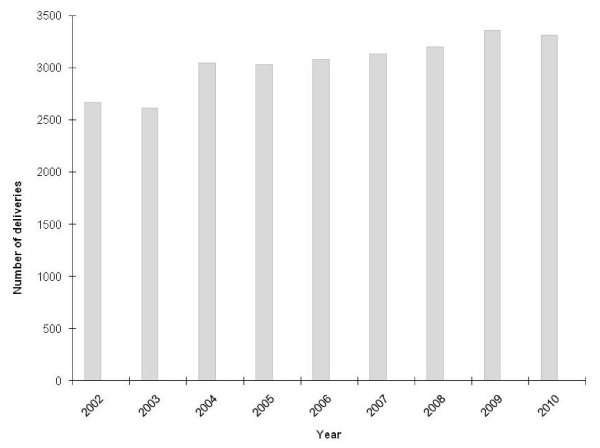
**Annual number of deliveries at Teule hospital**.

As an indicator of hospital utilization the annual number of deliveries from 2002 to 2010 was assessed (Figure 4). There were 2, 665 deliveries in 2002, which increased to over 3, 000 annual deliveries during 2004 to 2010.

## Discussion

There was an impressive decline in the incidence of malaria in the study site, which is consistent with previous reports from other locations [25-30]. From the first to the last year of surveillance, we noted an increase in the median age of children who were admitted with severe malaria, which supports the temporal reduction of malaria transmission in the area. A decrease in transmission intensity is known to be associated with an increase in the age of severe malaria cases [40,41]. A cluster-randomized survey conducted in Muheza in 2008 showed that the use of an insecticide-treated or any bed net among children less than five years of age was 54% and 71% respectively [42] but use of indoor residual spraying is not common in the community.

The decline of malaria in Muheza occurred in parallel with a reduction in invasive bacterial infections, particularly in non-typhoidal salmonellosis. Although over a shorter time period, our findings are similar to those from The Gambia, where the estimated incidence of NTS infection in Fajara fell from 60 (1979-1984) to 10 (2003-05) cases per 100, 000 person-years. In Basse, also The Gambia, the estimated incidence of NTS infection fell from 105 (1989-1991) to 29 (2008) cases per 100, 000 person-years [29]. In Kenya, from 1999 to 2002, there was a decrease in the number of paediatric admissions for malaria and for not only non-typhoidal salmonellosis, but other gram-negative bacteraemias [30]. In that study, the known protective effect of the sickle cell trait against malaria was used to evaluate whether the association between malaria and bacteraemia was causally related. Yearly case-control studies showed that the protective effect of sickle cell trait against bacteraemia waned as malaria rates declined confirming that malaria predisposes to bacteraemia. But the association between malaria and pneumococcal bacteraemia could not be assessed using the same method because sickle cell trait slightly increases the risk of pneumococcal disease (even without malaria). Thus, there is a strong suggestion that malaria increases the risk for some bacteraemias. More work needs to be done to understand this association.

That malaria increases the risk for bacteraemia is plausible for several reasons. The hemodynamic or inflammatory processes that occur during severe malaria can predispose to invasive bacterial infection. For example, malarial haemolysis may lead to impaired macrophage and neutrophil function, saturation of iron-binding proteins, and increased iron availability to NTS, a siderophilic organism [19]. Alternatively or in conjunction, sequestration of parasitized erythrocytes in post-capillary venules may affect the integrity of the bowel epithelium allowing transmigration of gram-negative bacteria. The authors are unable to explain why bloodstream infections with *E. coli*, a gram-negative organism ubiquitous in the bowel, did not decrease. The incidence of Hib and pneumococcal bacteraemia in the study site decreased over the relatively short period of four years. It is unlikely that these reductions were due to immunization, as neither Hib nor pneumococcal vaccines were widely available during the study period. Hospital-based surveillance data from Malawi showed a decline in the number of invasive pneumococcal infections from 2000 to 2009, which paralleled the scale-up of national antiretroviral therapy [43]. These trends are in contrast to The Gambia where the measured incidence of pneumococcal bacteraemia in Fajara and Basse did not fall. Over the four-year period, the number of typhoid fever cases in Muheza did not decrease. It is possible that there was an on-going typhoid fever outbreak during the third and fourth years of surveillance. However, in this population, the association of malaria with bacteraemia is probably most relevant in young children (among whom malaria occurs most frequently), whereas typhoid fever tends to occur more in the older age groups (Table 2).

There are several limitations of our study. First, the analysis used pooled data from three studies with slightly different enrolment criteria. To ensure consistent inclusion criteria in our analysis, the non-severely ill children in the first study were excluded from the analysis. The period from June 2007 to February 2008 had the highest percentage of children with severe malaria (55%) as the enrolment criteria during the drug trial was focused on signs of severe malaria and did not include neck stiffness, bulging fontanel and fever of three or more days. This more stringent enrolment criteria likely resulted in the failure to detect some clinically important bacteraemia cases, particularly typhoid fever, as reflected in the lowest incidence during the second year. But the overall trend showed a decrease in bacteraemias from the beginning to the end of surveillance. Second, the analytic plan was not developed *a priori*. Prospective studies on community-acquired bloodstream infections in sub-Saharan Africa are scarce [1] and the authors took advantage of this four-year surveillance dataset to explore potential associations. Third, it is possible that not all cases in the community were captured since detection was done using a passive hospital-based surveillance method. Actual disease burden may be higher. Fourth, with the exception of malaria and NTS, the numbers of bacteraemia cases used to calculate incidence were few and the duration of surveillance was limited to four years. Changes in incidence may be due to natural fluctuations in disease occurrence. Fifth, in this descriptive study, a causal relationship between the decline in malaria with that of NTS and possibly other invasive bacterial infections cannot be assessed. HIV infection, which is known to be associated with invasive NTS disease [19], as well as other bacterial infections, was not included in the analysis. The authors are not aware of any data suggesting changes in HIV/AIDS prevalence in the area during the study period. Sixth, it could be argued that other factors may have caused the decrease in bacteraemia incidence such as changes in health utilization behaviour. Alternative treatment options for fevers could have become available in the area during the study period. To the authors' knowledge, there were no such alterations in treatment options; changes in referral patterns of severe febrile illness are particularly unlikely. Although Mkinga district was formed, there has been no hospital established there and its health centres have approximately the same level of facilities and staffing as they did in 2005. The increasing number of deliveries at Teule indicates sustained hospital usage. Finally, it is possible that improvements in socio-economic status and living standards of the referral population caused the reduction in cases but such data is not available. It seems likely that there was a real decrease in malaria and bacteraemia rates during the study period.

These findings provide additional evidence for the association of malaria with bacterial disease. Strategies that prevent malaria also inhibit the risk of non-typhoidal salmonellosis and other bacteraemias. This has important implications for the impact of malaria control on all-cause child mortality. In Bioko there was a large drop in under-five mortality from 152 to 55/1000 births following malaria control [5]. In Zanzibar, the decline in falciparum malaria prevalence following the deployment of artemisinin-based combination therapy and distribution of long-lasting insecticidal nets was associated with a decrease in infant, child (aged 1-4 years) and under-five crude mortality by 33%, 71%, and 52%, respectively [6].

## Conclusion

From 2006 to 2010, a decline in malaria infections in Muheza was associated with a reduction in invasive NTS disease and other bacteraemias. Continued surveillance in Muheza would be important to understand longer-term trends.

## Competing interests

The authors declare that they have no competing interests.

## Authors' contributions

Conception, design and implementation of the studies: GM, BA, ICEH, AMD, LVS, HR, and JD. Laboratory supervision: BA. Data management: AM, DRK. Contributed to the data analysis: GM, BN, AM, DRK and JD. Participated in the writing and revision of the manuscript: GM, BA, BN, ICEH, AMD, RLO, JDC, LVS, HR and JD. All authors read and approved the final manuscript.
